# Perceived Control and Cognitive Function Among Acute Ischemic Stroke Patients in China

**DOI:** 10.1093/geroni/igab046.254

**Published:** 2021-12-17

**Authors:** Zhijian Liu, Xiangjing Kong, Jing Wang, Qin Wang, Bei Wu, Juan Li

**Affiliations:** 1 Department of clinical nursing School of Nursing Second Military Medical University, Shanghai, Shanghai, China (People's Republic); 2 School of Nursing Second Military Medical University, Shanghai, Shanghai, China (People's Republic); 3 Fudan University, shanghai, Shanghai, China (People's Republic); 4 Linyi People Hospital, Linyi, China, Linyi, Shandong, China (People's Republic); 5 New York University, New York, New York, United States

## Abstract

This study explored an association between perceived control and cognitive function among 437 acute ischemic stroke (AIS) patients in China. We collected data from one stroke center in each of the three cities (Shanghai, Nanjing, and Linyi) from June to December, 2020. Cognitive function was assessed by the Montreal Cognitive Assessment (MoCA), and perceived control was assessed by Perceived Control in Health Care Questionnaire at acute stage. Hierarchical linear regression was used. The average of perceived control and MoCA were 81.36±0.877 and 19.66±0.304, respectively. A number of 374 (85.6%) patients were in cognitive impairment and 63(14.4%) were cognitively normal. Perceived control was positively associated with cognitive function (β=0.103, p＜0.001). After controlling for stroke severity, age, gender and education, the association was still significant (β=0.041, p=0.014). These findings suggest that perceived control may be a potential target in cognitive interventions for AIS patients.

